# Delayed sequelae of a proximal tibial fracture presenting with advanced arthritis managed by total knee arthroplasty

**DOI:** 10.11604/pamj.2025.52.92.49358

**Published:** 2025-11-03

**Authors:** Anjali Nawkhare, Mitushi Deshmukh

**Affiliations:** 1 Department of Musculoskeletal Physiotherapy, Ravi Nair Physiotherapy College, Datta Meghe Institute of Higher Education and Research, Sawangi (Meghe), Wardha, Maharashtra, India

**Keywords:** Total knee arthroplasty, sequela of proximal tibial fracture, tibial plateau, orthopaedic surgery

## Image in medicine

Six (6) years after receiving surgery for a compound grade 3B fracture of the proximal right tibia, a 45-year-old man with a history of bronchial asthma came with chronic right knee pain and limited mobility. Magnetic resonance imaging (MRI) showed that the tibiofemoral and patellofemoral joints had diminishing articular cartilage and signs of a chronic cleavage fracture affecting both tibial plateaus. Along with a degenerative tear of the posterior horn, degenerative alterations were observed in both horns and the lateral meniscus body. Radiographs showed subchondral sclerosis and narrowing of the joint space, which are post-traumatic osteoarthritic alterations. The patient underwent successful total knee arthroplasty (TKA), leading to marked improvement in joint function and pain relief. The patient had a successful functional recovery following a total right knee replacement. This case emphasizes the long-term degenerative effects of high-grade open tibial plateau fractures and how total knee replacement can help restore joint function in these complex situations.

**Figure 1:**
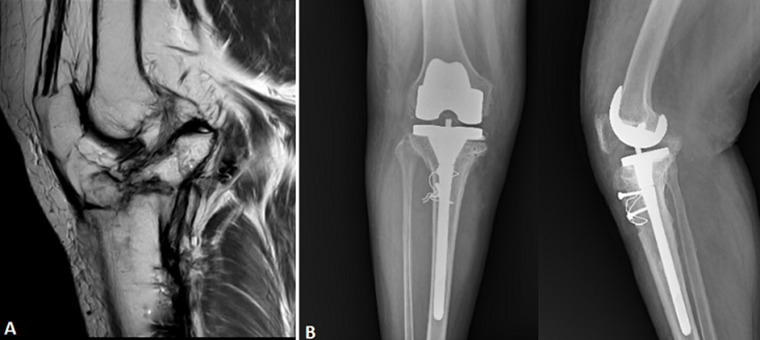
A) magnetic resonance imaging of the right knee demonstrating chronic post-traumatic changes, including thinning of the articular cartilage, degenerative tear of the lateral meniscus, and evidence of an old cleavage fracture involving both tibial plateaus; B) radiographs showing postoperative anteroposterior and lateral views of the right knee showing a well-aligned total knee replacement (TKR) implant with femoral component and tibial stem extension; the presence of cerclage wires and screws indicates prior fixation, consistent with previous management of a complex tibial plateau fracture

